# Full-Length Structure and Heme Binding in the Transcriptional Regulator HcpR

**DOI:** 10.1021/acsomega.5c01735

**Published:** 2025-12-09

**Authors:** Benjamin Ross Belvin, Faik N. Musayev, Carlos R. Escalante, Janina P. Lewis

**Affiliations:** † The Philips Institute for Oral Health Research, Virginia Commonwealth University, Richmond, Virginia 23298, United States; ‡ Department of Medicinal Chemistry, 6889 Virginia Commonwealth University, Richmond, Virginia 23298, United States; § The Center for Drug Discovery, Virginia Commonwealth University, Richmond, Virginia 23298, United States; ∥ Department of Cellular, Molecular and Genetic Medicine, Virginia Commonwealth University, Richmond, Virginia 23298, United States; ⊥ Department of Microbiology and Immunology, Virginia Commonwealth University, Richmond, Virginia 23298, United States

## Abstract

HcpR is a heme-dependent transcriptional regulator present in many Gram-negative anaerobic bacteria. In the perio-pathogen *Porphyromonas gingivalis*, HcpR is crucial for the response to reactive nitrogen species such as nitric oxide (NO). Binding of NO to the heme group of HcpR leads to transcription of redox enzyme Hcp. However, the molecular mechanisms of binding of heme to HcpR remain unknown. In this study, we present the 2.3 Å structure of the *P. gingivalis* HcpR. Interdomain interactions present in the structure help to form a hydrophobic pocket in the N-terminal sensing domain. A comparison analysis with other CRP/FNR-family members reveals that the molecular mechanisms of HcpR-mediated regulation may be distinct from those of other family members. Using docking studies, we identified a putative heme-binding site in the sensing domain pocket. *In vivo* complementation and mutagenesis studies verify one pocket residue, Met68, as an important reagent in HcpR-mediated transcriptional activation. Finally, heme-binding studies with purified forms of recombinant HcpR support Met68 as a crucial residue for heme binding as well as coordination.

## Graphical abstract

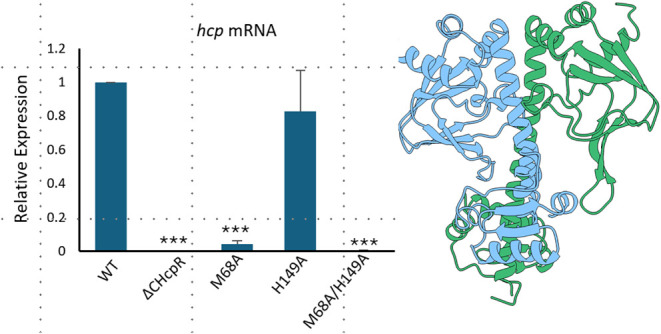

## Introduction

HcpR is a sensor-transcriptional regulator of nitrosative stress response. Found in many Gram-negative anaerobic bacteria, it regulates the expression of *hcp*, an enzyme crucial for survival under nitric oxide (NO) and other forms of nitrosative stress.^1,2^ HcpR belongs to the cAMP receptor protein (CRP)/fumarate and nitrate reduction regulatory protein (FNR) (CRP/FNR) family of regulators. The family is named after the first two regulators identified but includes members regulating various sets of genes in response to different environmental signals. The CRP family of proteins includes the catabolite gene activator protein (CAP) and the CO-sensing protein CooA.^3,4^ This large family senses changes in the intracellular environment by binding effector molecules that allosterically activate the protein for DNA binding.^5^ The CRP family contains a significant number of subfamilies that have evolved to sense a wide variety of effector molecules and utilize different cofactors.^5^ One of these subfamilies, the DNR-HcpR group, has evolved to sense NO using metal cofactors such as heme and is phylogenetically distinct from other heme-containing CRP family members such as CooA.^6^ Although not all HcpRs have been demonstrated to bind heme, a growing subset have been characterized as heme-binding proteins.^7^

HcpR is crucial for the survival of the periodontal pathogen *Porphyromonas gingivalis*.^8^ In the periodontal pocket, *P. gingivalis* is exposed to numerous nitrosative stresses, including immune cell-derived NO as well as elevated levels of nitrite from saliva.^9,10^ Loss of *hcpR* or *hcp* sensitizes the bacteria to physiological concentrations of nitrite and NO, causing decreased survival with host cells and *in vivo* using mouse models.^8,11–13^ Previously, we have demonstrated that HcpR from *P. gingivalis* can sense nanomolar concentrations of NO and upregulates the expression of *hcp* in response to these stresses.^14^ Furthermore, we have shown that binding of HcpR to the *hcp* promoter depends on heme.^8^

In heme-based sensor proteins, the heme iron complex serves as the binding site for gaseous molecules such as NO, CO, or O_2_. Displacement of an axial side chain coordinating the heme iron is often utilized for allosteric activation of heme proteins.^15,16^ In HcpR, resonance Raman spectroscopy reveals that the heme exists in a 6-coordinate system. Addition of NO transitions the system to a 5-coordinate, displacing both axial side chains of HcpR.^14^ The capability of NO to displace the proximal side chain is a distinctive facet of the NO-heme interaction that is not typically seen in CO or O_2_ heme sensor proteins.^16^ The 5-coordinate NO-bound heme in NO sensor proteins such as HcpR and DNR distinguishes it from the CO sensor CooA, which is a 6-coordinate system in the CO-bound state and is not activated by NO.^17–19^ The different nature of the gas-binding properties has implications for the mechanism of NO-dependent activation in HcpR.

The identity of the axial side chains in HcpR remains unknown, as does the nature of heme binding to HcpR (the location of the exact heme-binding pocket). Previously, we solved the structure of the sensing domain of HcpR (ΔCHcpR), which lacks the (highly flexible) C-terminal DNA-binding domain.^14^ While the truncated structure confirms the inclusion of HcpR as a CRP-family regulator, information on interdomain interactions and how this may influence the heme-binding nature of the protein was lost. In this study, we present the structure of full-length HcpR (FL-HcpR). Using a docking approach supported by site-directed mutagenesis and heme-binding studies, we identify a putative heme-binding site and important residues in heme coordination. A comparison of HcpR with the structures of CooA and CAP reveals a novel mechanism that may control the NO-mediated activation of HcpR.

## Materials and Methods

### Generation of Recombinant Proteins

The full-length HcpR-encoding gene (PG1053) was cloned into the pET21d vector at the *Bam*HI and *Xho*I sites using gene-specific primers with complementary restriction site overhangs. Vector and PCR fragments were cleaved, purified, ligated, and transformed into DH5α *E. coli* for screening. Positive clones were identified, and whole plasmid sequencing was subsequently performed to confirm the proper genetic content. The recombinant *hcpR-*pET21 vector was transformed into BL21­(DE3) cells (New England Biolabs) for inducible protein expression.

For recombinant HcpR expression, overnight cultures were used to inoculate 1 L of LB broth. Cultures were grown at 37 °C with antibiotics (50 μg/mL carbenicillin) and shaking until cultures reached mid-log phase (OD_600_ = 0.5–0.7), and expression of HcpR was induced using 1 mM IPTG overnight at 37 °C. HcpR was affinity purified using Ni-NTA agarose (Qiagen) or TALON Cobalt resin (Thermo Fisher). Briefly, cells were lysed using CellLytic B reagent (Millipore-Sigma), and cell supernatant was added to the column containing metal affinity resin. Columns were washed with a 50 mM Na_2_H_1_PO_4_, 300 mM NaCl, 20 mM imidazole, pH 8.0 buffer. For elution, imidazole concentration was increased to 250 mM. For crystallization, HcpR was further purified via gel exclusion chromatography on a Superdex 75 column in crystallization buffer (25 mM Tris-HCl, 150 mM NaCl, 1 mM TCEP, pH 7.4). The protein was stored in this buffer at 4 °C until setting of the crystallization plates. Purification of recombinant mutant HcpRs was performed identically to the wild-type HcpR.

For His-tag removal and heme reconstitution, purified HcpR was concentrated and desalted into a 25 mM phosphate, 150 mM NaCl, and 1 mM TCEP pH 7.4 buffer using a PD-10 desalting column. The His-Tag was removed by incubating with TEV protease at 4 °C for 24–48 h before passing through a Ni-NTA agarose column to remove cleaved His-Tag, TEV protease, and undigested HcpR. A 1.1 molar excess of heme was added to purified HcpR and allowed to incubate overnight at 4 °C in the dark. Unbound heme was removed by overnight dialysis at 4 °C or buffer exchange using a desalting column. Heme was prepared fresh from hemin powder (Sigma-Aldrich) solubilized in 1 M NaOH and then diluted to 0.1 M NaOH for a final concentration of ∼1 mg/mL heme. The heme concentration was confirmed using the pyridine hemochrome assay.^20^ The efficiency of heme reconstitution of HcpR was measured by using the pyridine hemochrome assay.

### Crystallization and Structure Determination

Freshly prepared HcpR (15.1 mg/mL) in 25 mM Tris-HCl, 150 mM NaCl, 1 mM TCEP, pH 7.4 was used to screen for initial crystallization conditions using a wide range of commercially available screening kits with the Gryphon crystallization robot (Art Robbins) employing the sitting-drop vapor-diffusion method at 20 °C. 0.3 μL of protein solution and 0.3 μL of reservoir were mixed to equilibrate against 60 μL of reservoir solution. Crystals appeared in 1 week under several conditions (K/Na tartrate, Na formate, Na citrate tribasic, and PEG-3350) at a pH range of 4.6–6.5. The hanging-drop vapor-diffusion method was used to improve the quality and size of the crystals in 24-well VDX crystallization plates. A 5 μL droplet consisting of a 1:1 protein:reservoir solution was equilibrated against 800 μL of reservoir solution at 20 °C. The high-diffraction-grade single crystal of wild-type HcpR crystallized at low pH (4.6). The crystallization conditions were 0.1 M sodium acetate, pH 4.6, and 1.8 M sodium formate. The crystals diffracted up to 2.3 Å resolution and belonged to space group P4_1_2_1_2 with unit cell dimensions of *a* = *b* = 106.28 Å, and *c* = 116.77 Å. Crystals were cryoprotected by transfer into the mother liquid solution with 5% glycerol, and the glycerol concentration was then slowly increased to 20% (v/v). Subsequently, the crystals were flash-cooled in a cryogenic nitrogen stream. X-ray diffraction data were collected at 100 K using a Rigaku MicroMax-007 HF X-ray generator and an EIGER R 4 M detector. The total number of 0.5° oscillation frames was 664, but only 150 frames were used for the final data processing and reduction because the observed diffracted intensity and its resolution decayed from radiation damage during data collection. Effects of both radiation damage and nonuniform irradiation on measured intensities and the structure factors derived from them, resulting in evaluated values for R_work_ and R_free_ in the highest-resolution shells. The data was processed with *CrysAlis* ^*Pro*^ 40.64.69a (Rigaku) and the CCP4 suite of programs.^21^

### Model Building and Refinement

The crystal structure was solved with *Phaser* in the *Phenix* software package using the sensing domain of HcpR (PDB entry: 6NP6) as the search model.^14,22,23^ Automodel building resulted in an initial model of 572 amino acid residues, built with R_work_ of 28.21% and R_free_ of 31.71%. The structure was refined using Phenix software, along with manual model building in the graphics program Coot.^24^ After several cycles of refinement and manual model building, the electron density belonging to the C-terminal domain became visible. It was possible to fit the helix-turn-helix motif of the DNA-binding domain. The structure was refined to 2.3 Å resolution with a final R_work_ of 21.5% and R_free_ of 25.56%.

### Docking Studies

Structural models of the heme group and the FL-HcpR dimer were prepared for docking calculations using the Graphical User Interface AutoDock Tools (ADT). The grid size was set to 40 × 40 × 40 *xyz* points with grid spacings of 0.375 Å, and the grid center was placed at *x* = 111.826, *y* = 42.032, *z* = 151.488. Other parameters were maintained under the default configuration. The two .pqt files were imported into AutoDock Vina based on a fixed geometry of HcpR and heme.^24^ The result with the lowest energy of binding (−8.3 kcal/mol) was selected for further analysis in ChimeraX.

### UV–Vis Spectrum Studies

Spectra of heme-reconstituted HcpR was recorded using a Genesys 150 UV–vis spectrophotometer (Thermo Fisher) in a gastight 1 cm quartz cuvette in 25 mM sodium phosphate, 150 mM NaCl, 1 mM TCEP, pH 7.4 buffer. To obtain anaerobic samples, reconstituted HcpR was placed in an anaerobic chamber (Coy Laboratories) overnight at 4 °C in an atmosphere of 80% N_2_, 10% CO_2_, and 10% H_2_. The ferrous form of heme was obtained using sodium dithionite. A fresh 1 M stock solution of dithionite was prepared in buffer and then added directly to the protein sample to achieve a final concentration of approximately 25 mM.

UV–vis spectra of the nitrosylated HcpR were obtained by the addition of DETA-NONOate (Cayman Chemical) to anaerobic, reduced HcpR. NONOate was dissolved in anaerobic, deoxygenated buffer, and the concentration was measured by UV absorbance at 252 nm (ε = 7640 M^–1^ cm^–1^). NONOate solution was added to the protein samples at approximately 100 molar excesses. The samples were incubated for 10 min before UV–vis spectra were assessed.

### Bacterial Strains and Growth Conditions

*P. gingivalis* W83 (strain V2802) was maintained in an atmosphere consisting of 80% N_2_, 10% CO_2_, and 10% H_2_ in an anaerobic chamber (Coy Laboratories). The *hcpR*-deficient *P. gingivalis* (V2807) was derived previously.^8^ Strains were grown on TSA-blood agar plates (Tryptic Soy Agar, 5% sheep blood—BD Biosciences) or in tryptic soy broth (TSB—BD Biosciences) supplemented with hemin (5 μg/mL) and Vitamin K (1 μg/mL). Plasmid-containing strains were maintained with tetracycline (0.5 μg/mL).

### Cloning and Site-Directed Mutagenesis of *P. gingivalis* *hcpR* Complement Plasmids

The *P. gingivalis* *hcpR*-deficient strain (V2807) was complemented using the pG108 plasmid.^25^ A copy of the *hcpR* gene was synthesized downstream of the *ermF* promoter for constitutive expression and cloned into the pG108 vector at the *Sph*I and *Bam*HI restriction sites to create the pG108-*hcpR* plasmid. The QuikChange II XL mutagenesis kit (Agilent) was used to introduce mutations at Met68, His149, and L156 to convert them to Ala (Met68, His149) or a stop codon (L156). Primers used for this study: M68AF-CCGGAAGGCCCCACCGCCTCAGCACGAATCTC; M68A-R-GAGATTCGTGCTGAGGCGGTGGGGCCTTCCGG; H149AF-CGCAAGC TGAGCTGAGCGATTTTCTTCAT; H149A-R-GTGCTTTCCTGATGAAGAAAATCGCTCAG. Sequencing-verified clones were used to complement the *hcpR*-deficient V2807 strain.

### Electroporation and Complementation of HcpR-Deficient *P. gingivalis*

Fresh bacterial cultures on blood plates were used to inoculate 5 mL of Tryptic Soy broth culture overnight at 37 °C. The next day, this culture was diluted 1:10 with fresh media and grown to mid-log phase (0.5–0.6 OD_600_). Cells were harvested by centrifugation at 8000 × *g* for 15 min at 4 °C, washed in 25 mL cold electroporation buffer (10% glycerol, 1 mM MgCl_2_), and suspended in ∼250 μL EP buffer. To a prechilled 0.2 cm electroporation cuvette, 50 μL of washed cells and 1–2 μg of pG108-*hcpR* plasmid DNA were added. The cuvette was placed in an electroporation chamber and pulsed using settings at 2.5 kV, 5 ms, and 400 Ω on a Bio-Rad Gene Pulser II. Immediately after electroporation, 500 μL of prewarmed media was added, and cells were grown overnight in the anaerobic chamber. The next day, the bacteria were plated on blood agar plates supplemented with 0.5 μg/mL tetracycline. Colonies that appeared after 5–6 days of incubation were replated and screened for the proper genetic content.

### Nitrite Growth Studies

Overnight cultures of plasmid-complemented Δ*hcpR* *P. gingivalis* started from blood agar plates were diluted to an OD_600_ of 0.05. These cultures were supplemented with 0, 1, or 2 mM sodium nitrite and grown overnight anaerobically at 37 °C. The next day, the OD_600_ was measured to assess growth of the*P. gingivalis* strains.

### qRT-PCR

To test HcpR function *in vitro,* *P. gingivalis* V2807 complemented with plasmids containing recombinant *hcpR* were grown to mid-log phase in Tryptic Soy broth and then exposed to 0.2 mM nitrite for 15 min. The bacterial cells were harvested, RNA was purified using Zymo Quick-RNA mini-prep kit and treated with DNA-free DNase kit (Thermo Fisher), and cDNA was generated using High-Capacity cDNA reverse transcriptase kit (Thermo Fisher). Real-time qPCR analysis was performed with a SYBR green-based detection system on a QuantStudio 3 thermal cycler. Expression levels of *hcp* were normalized to the levels of the Pg16s rRNA. Primers used for qPCR: hcp-F: AAAGCTGTCATCGTCCTGCT; hcp-R: CGATCAGCGTCCGAATATCT; Pg16s-F: AGGCGGAATTCGTGGTGTAG; Pg16s-R: TTTGATACCCACGCCTTCGT.

### Bioinformatics

Molecular graphics and analyses were performed with UCSF ChimeraX, developed by the Resource for Biocomputing, Visualization, and Informatics at the University of California, San Francisco.^26,27^ ChimeraX was used to superimpose structures and determine distances, solvent-excluded areas, and any potential clashes/contacts. AlphaFold 3 was used to model the DNA-binding domains in the active form bound to DNA.^28^ Sequence alignments were constructed using Clustal Omega.^29^ Alignments were portrayed using Jalview.^30^ Structure-based alignments were constructed using TM-align.^31^

## Results

### Overview of the Structure of Full-Length HcpR

Crystals of FL-HcpR belonging to space group P4_1_2_1_2 diffracted to 2.3 Å. The structure was solved by molecular replacement using the truncated HcpR structure (PDB ID: 6NP6). The asymmetric unit contains one homodimer, comprising chains A and B. There was no significant loss of electron density along the backbone of the structure, and all 228 amino acids are visible in chain A. In chain B, there is a one-residue gap due to lack of electron density. In subunit A, the uncleaved cloning tail containing polyhistidine and TEV sites can also be observed on the map (Figure S2A). The data collection and refinement statistics are summarized in Table 1.

**Table 1:** Data Collection and Refinement Statistics

Data Collection Statistics	
Space group	P4_1_2_1_2
Unit-cell *a, b, c* (Å)	106.28, 106.28, 116.77
Resolution (Å)	26.57–2.30 (2.38–2.30)
Total reflections	161187 (15780)
Unique reflections	29963 (2938)
Redundancy	5.4 (5.4)
Completeness (%)	99.0 (100.0)
Average I/σ(I)	24.6 (3.0)
R_merge_ (%)^a^	5.3 (53.7)
Refinement Statistics	
Resolution (Å)	26.57–2.30 (2.37–2.30)
No. of reflections	29903 (2706)
R_work_ (%)	21.50 (25.36)
R_free_ (%)^b^	25.56 (29.81)
RMSD bonds (Å)	0.007
RMSD angles (°)	1.04
Ramachandran Plot Quality	
Most favored (%)	98.25
Allowed (%)	1.75
Disallowed (%)	0
Average B (Å^2^)/atoms	
All atoms	62.47
Protein	63.14
Additives	53.18
Solvent	50.84
**Number of non-hydrogen atoms**	3673
Macromolecules	3467
Additives	24
Water	182
Protein residues	465

a R_merge_ = Σ_*hkl*_Σ_i_|*I*_i_(*hkl*) – <*I*(*hkl*)>|/Σ_*hkl*_Σ_i_ *I*_i_(*hkl*).

b R_free_ was calculated from 5% randomly selected reflection for cross-validation. All other measured reflections were used during refinement.

Each HcpR monomer comprises an N-terminal ligand-sensing domain (SD) and a C-terminal DNA-binding domain (DBD) (Figure 1A). In agreement with our previous work, HcpR forms an ∼48 kDa homodimer. The SD encompasses residues M1-L151, while the DBD extends from residues L156-E228, forming a winged helix-turn-helix motif (Figure 1A). The dimerization helices (helix E) of each monomer form a leucine zipper motif along residues P127-L151, with interface interactions between symmetry-related Leu, Ile, and Met residues stabilizing the formation of the homodimer with a buried solvent-excluded area of 538.13 Å^2^ (Figure 1B). The leucine zipper and DBD are connected by residues spanning S152-L156, known as the “hinge”. This is a metamorphic region that breaks helix E in chain A, forming a loop that connects the SD and DBD, but forms part of an extended helix E in chain B, spanning residues 127 to 171 (Figure 1C). The electron density of this loop is well-defined in chain A of the structure (Figure S2B). The flexible nature of the hinge is crucial to the mechanisms that govern the DNA binding of many CRP family regulators.^32^

**Figure 1:**
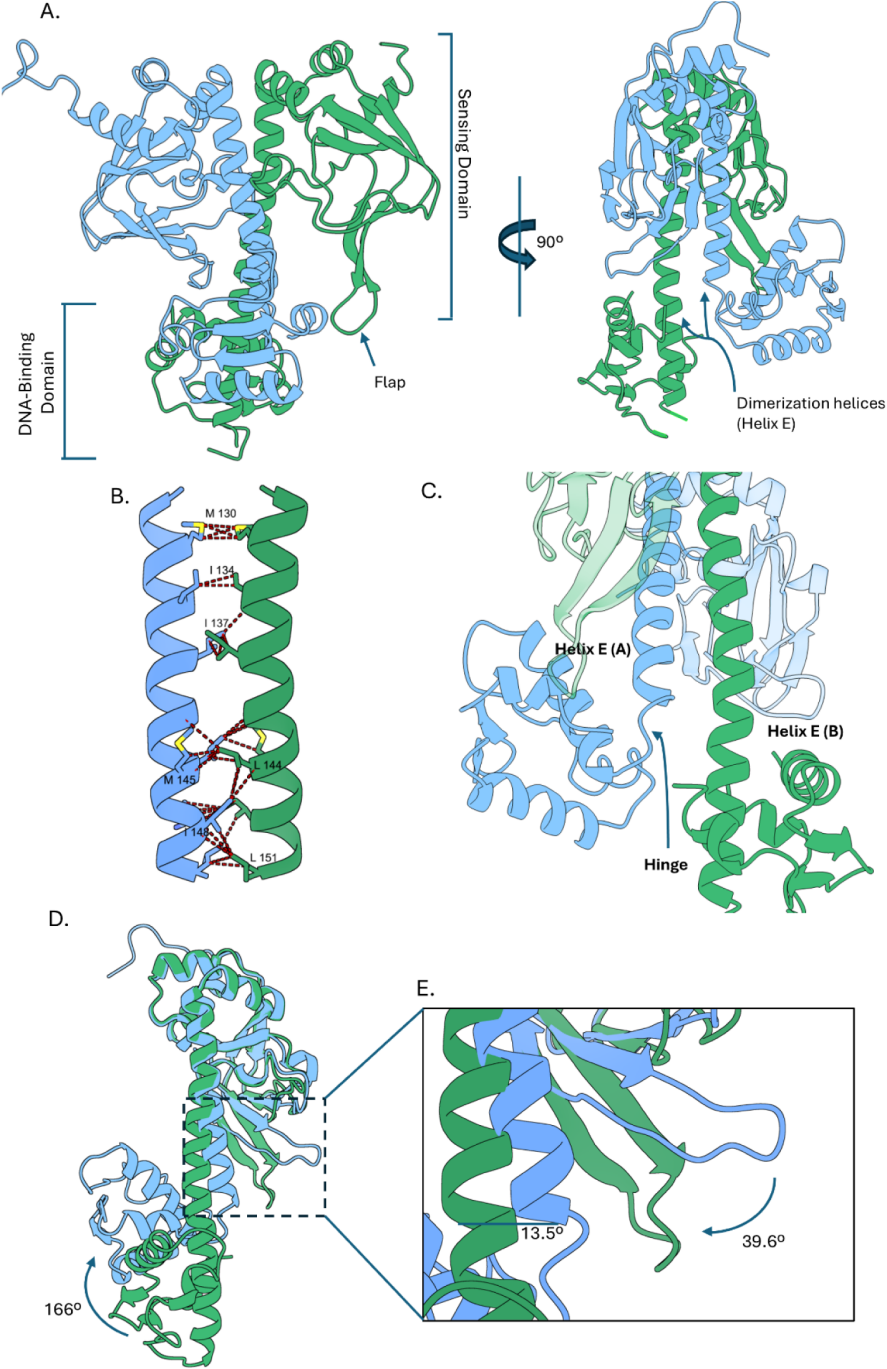
Overview of the crystal structure of full-length HcpR. (A) Ribbon diagram of the HcpR shown in forward and 90° side views showing the orientation of the N-terminal sensing domain and C-terminal DNA-binding domain. Chain A is colored in blue, and Chain B is colored in green. The two chains form a homodimer. (B) Dimerization helices of HcpR. The homodimer is stabilized by hydrophobic interactions among symmetry-related Met, Leu, and Ile residues. Dashes indicate Van der Waals contacts between symmetry-related residues. (C) Highlight of the asymmetry of the DNA-binding domains of chains A and B. Helix E of chain A is broken at the hinge region at residue 152, where Helix E of chain B is extended through residue 170. (D) Superimposing chains A and B highlights the asymmetry of the DNA-binding domains. The DBD of chain A is rotated 166° with respect to chain B. (E) Inset of the boxed region in panel (D). The flap region of chain B is angled backward 39.6° to create a pocket in the SD of chain B.

The sensing domain of HcpR comprises 7 β-strands and 4 α-helices. The β-strands form a “jelly roll” like fold typically found in the CRP family of regulators. The standard CRP-family topology includes 8 β-strands organized in an antiparallel fashion, but in HcpR the sixth β-strand is completely distorted by a proline residue (Pro89), creating an extended loop in the β6-β7 turn with a 1-turn helix. This extended loop region thereby influences the potential size and location of the ligand-binding pocket via partially disrupting the hydrogen bond matrix between the β-strands. Pro89 and the region surrounding it are highly conserved in the HcpRs from*Porphyromonas* and*Prevotella* species (Figure S1A), suggesting it plays a pivotal role in shaping the heme-binding pocket in HcpR and its homologues.

Superimposing the two HcpR chains reveals three structurally dissimilar regions. The DNA-binding domain of chain A is rotated by almost 166° relative to chain B (Figure 1D). One subunit is in an apparent “OFF” state (chain B—green) with the recognition helix folded in toward the protein, while the other is in an apparent “ON” state (chain A—blue) with the recognition helix exposed. The second difference is in the flap region, with a 39.6° rotation between chains (Figure 1E). This is generated by the interaction between the β-hairpin of chain A and the DBD of chain B, particularly an interaction between R226 and main chain carbonyl oxygens from V69 and G70 (Figure S2C). The final difference is in the second half of the leucine zipper, where they superimpose, forming a 13.5° angle (Figure 1E).

### Comparison to Other CRP-Family Regulators

We conducted a structural comparison with the CRP family member CooA in complex with heme to identify a potential heme-binding pocket in HcpR (Figure 2A). Our previous ΔCHcpR sensing domain structure identified two hydrophobic pockets, but they were not large enough to accommodate a heme molecule.^14^ HcpR represents a distinct subgroup in the phylogeny of the CRP family, and this is present in both a structural sequence of CooA and HcpR. The two proteins share a conserved secondary structure and domain architecture despite superimposing with an RMSD of 7.60 Å (across the complete 200 C-α atom pairs of CooA). However, by just aligning the sensing domains, the proteins superimpose with an RMSD of 1.19 Å (Figure 2A). In CooA, the heme-binding pocket lies at the top half of the leucine zipper α-helices, with the heme coordinated by the N-terminal Pro2 of one subunit and His77 of the second subunit (Figure 2A). In HcpR, this position is occupied by the extended β6-β7 loop (Figure 2A inset right). This extension is also evident in the sequence alignment of HcpR and CooA (Figure S3A). Moreover, the structural alignment reveals that the heme-coordinating residues of CooA are not conserved in similar locations in HcpR (Figure S3B), suggesting that the heme-binding site in HcpR differs from that of CooA. This is also evident by the disorganized nature of the CooA N-terminus, allowing the N-terminal residue of one subunit to coordinate heme in the binding pocket of the opposite subunit. By comparison, the N-terminus of HcpR is highly organized and oriented away from the protein, making it unable to coordinate the heme (Figure 2A inset left).

**Figure 2:**
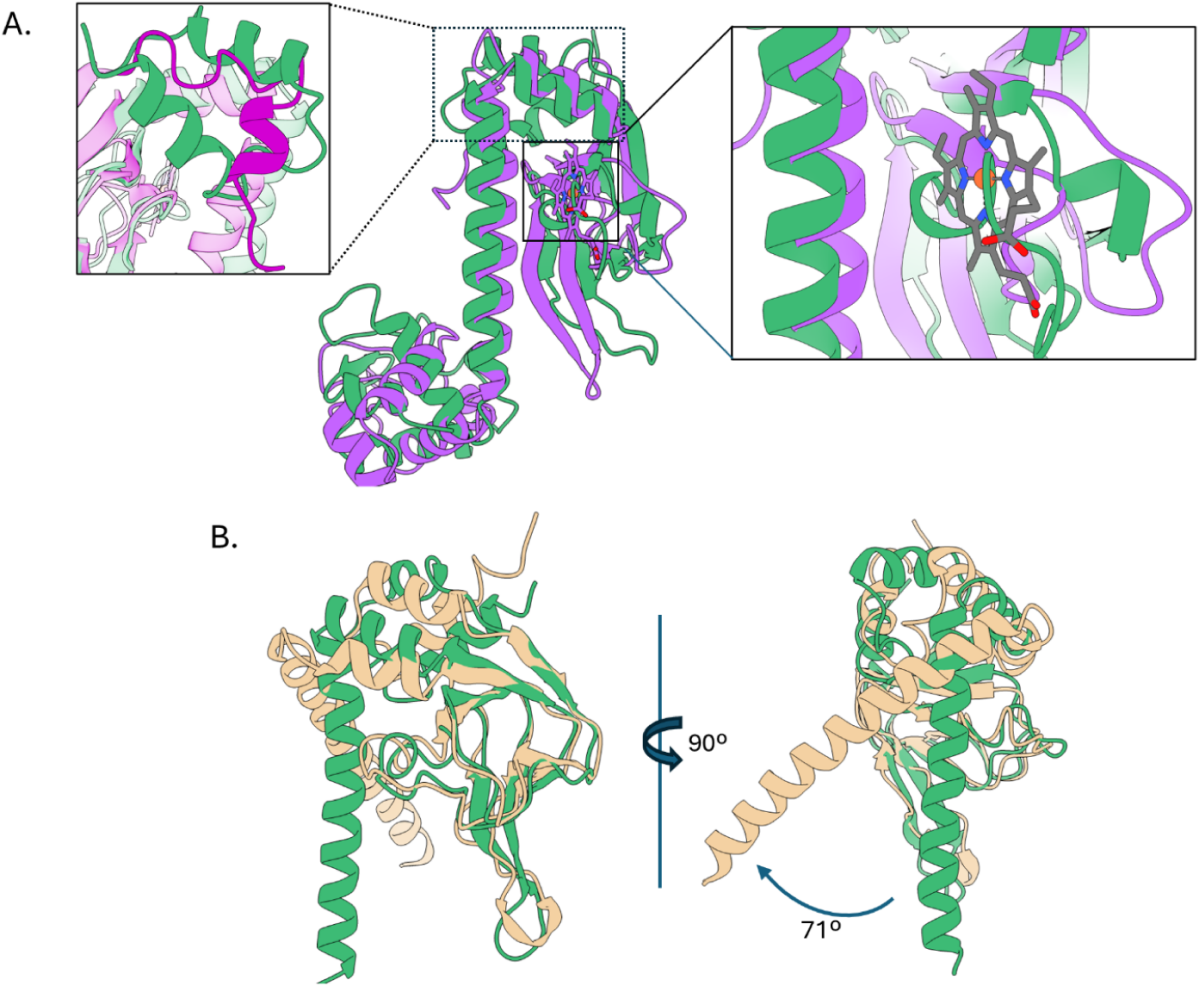
Comparison and conformational differences between HcpR, CooA, and DNR. (A) Overlay of the sensing domains of HcpR chain A (green) and CooA chain A (purple—PDB ID 1ft9) depicting the organization of each protein. The N-terminal domain of HcpR (extending from residues 1–127) is slightly larger than that of CooA (extending from residues 2–107). Inset left: The N-terminus of CooA is unstructured to allow the N-terminal Pro residue to coordinate heme. By comparison, the N-terminus of HcpR is well organized. Inset right: The extended loop region represented by residues 89–94 in HcpR occupies the heme-binding site of CooA. (B) View of the sensing domains of HcpR (green) and DNR (tan—PDB ID 3dkw). The dimerization of DNR is at a 71° difference when compared to HcpR.

DNR, a CRP family protein found in*Pseudomonas*that also uses heme to sense NO, shares a 20% sequence identity with HcpR and a high sequence similarity (Figure S3C). However, its heme-bound structure is not known.^33,34^ In the full-length DNR crystal structure, both monomers crystallize with an extended dimerization helix, resembling chain B in FL-HcpR, with no contacts observed between the sensing and DNA-binding domains. Comparing HcpR and Apo-DNR reveals a similar structural organization (Figure 2B), with sensing domains superimposing with an RMSD of 1.29 Å. Much like HcpR, the sixth β-strand of DNR’s sensing domain is partially distorted by a helical turn, altering the putative heme-binding site. However, in the absence of SD-DBD interactions, the current crystal structure of DNR with both chains in the “OFF” conformation lacks a pocket large enough to accommodate the heme molecule. DNR’s flap region appears to be extended out perpendicularly from the protein, an orientation that appears to be stabilized by crystal packing forces. This is most drastically seen in the differences in the angles of the dimerization helices when the sensing domains are overlaid. There is a 71° difference in the angle of the helix when compared with HcpR (Figure 2B).

Comparison with CAP reveals that one of the hydrophobic pockets we identified in our previous ΔCHcpR structure and the CAP cyclic-AMP binding sites are situated in close but not identical positions (Figure S4A). In HcpR, the extended loop region partially occupies the cyclic-AMP binding site in CAP (Figure S4B). Furthermore, CAP’s pocket volume is smaller to accommodate the smaller ligand cAMP. Taken together, we conclude that the presence of the long β6-β7 loop suggests that the heme-binding pocket in HcpR must be located in a different position.

### HcpR Heme Binding Pocket

Resonance Raman spectroscopy revealed that heme-bound HcpR is primarily in a 6-coordinate state, with one of the axial bonds being weaker and easily broken.^14^ To identify potential heme-binding sites, we used AutoDock Vina with the dimer structure as the target.^24^ Results show that one pocket formed between the DBD of chain A and the flap of the SD from chain B was the most significant, as six of the highest-scoring poses positioned the heme group around this location (Figure 3A, Figure S5A,B). The highest-scoring hit identified Met68 and His149 as potential heme-coordinating residues (Figure 3B). Met68 is highly conserved in HcpR sequence alignments (Figure S1). Three of the other poses are simply rotations of the heme around the axis that is perpendicular to the heme plane. A second pocket was identified on chain A at a site located between the leucine zipper α-helix and the β-sheet of the SD; however, there are no residues coordinating with the heme’s iron in the vicinity (Figure S5B). In addition to the coordination of heme iron, several residues from both chains in and around the pocket are positioned to potentially form contacts with the heme. These include hydrophobic interactions from Val69, Ile78, Pro71, Pro100, Val101, Met145, and Ile148, and hydrogen bonding and ionic/salt bridge interactions between the acid groups of heme and Lys146, Ser225, and Arg226 (Figure 3C). Some of these residues are conserved (Ile78, Pro100, Lys146) in HcpR and its homologues, with the others sharing a high degree of similarity in the sequence alignments (Figure S1A). In the crystal structure, this pocket is occupied by a portion of the polyhistidine tail and the TEV protease site that traverses across a tunnel created by the flap of chain B and the DBD of chain A (Figure S2A). The hydrophobic character of the pocket likely induced the tail interaction and stabilized the “ON” conformation.

**Figure 3:**
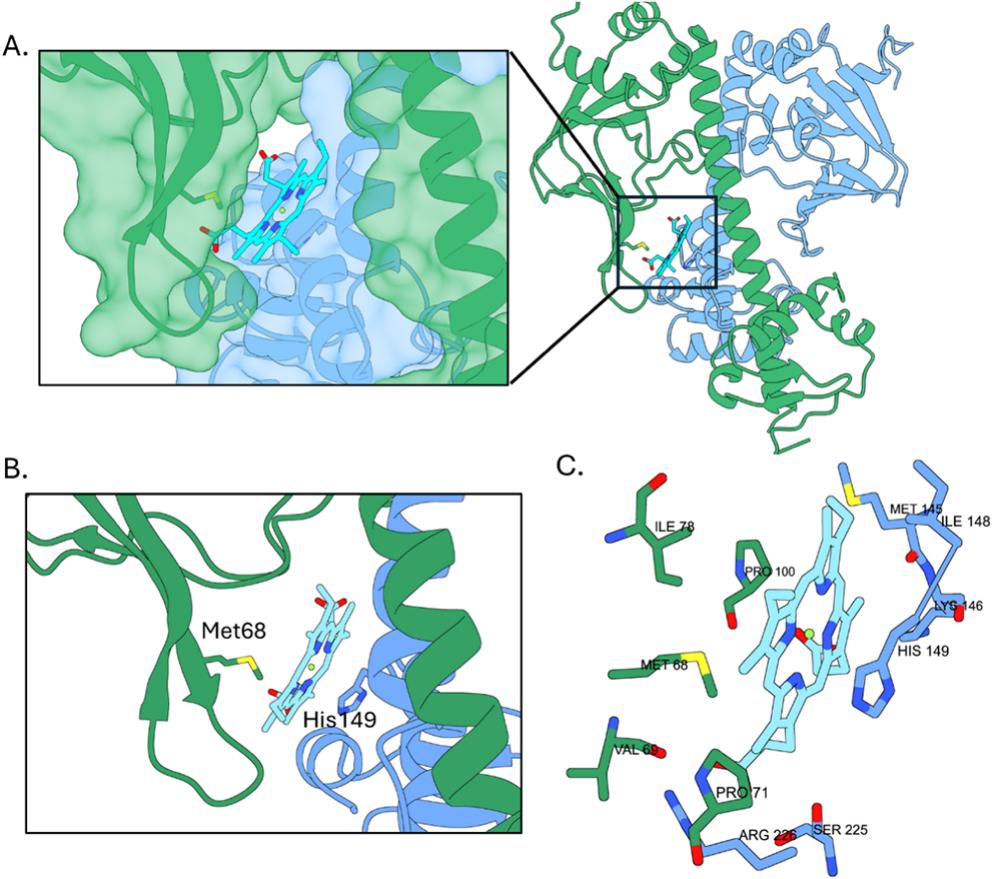
Heme docks into the hydrophobic pocket of HcpR. (A) Overview of heme docking in the HcpR pocket. Heme docks at the interface between chain B SD and the middle portion of helix E of chain A. (B) Side chains present in the putative heme pocket. Both Met68 and His149, residues commonly implicated in heme coordination, are optimally located in the pocket to coordinate the heme iron. (C) Side chains present in the heme pocket that form potential contacts with docked heme.

To determine the importance of Met68 or His149 for HcpR activation, we generated alanine mutations and complemented the*P. gingivalis* *hcpR-*deficient strain (V2807).^8^ Since *P. gingivalis* lacking HcpR does not grow in physiologically relevant concentrations of NO_2_^–^, a strain with a deficient HcpR cannot tolerate elevated NO_2_^–^ levels. Treatment with NO_2_^–^ reveals a significant decrease in the growth of the M68A strain at 1 mM NO_2_^–^ and no growth at the higher concentration of 2 mM (Figure 4A). The double mutant of M68A/H149A could not grow at either concentration of NO_2_^–^ and was comparable to the ΔCHcpR negative control, which completely lacks the DNA-binding domain. Unexpectedly, the H149A mutant saw no significant decrease in growth at either concentration of NO_2_^–^.

**Figure 4:**
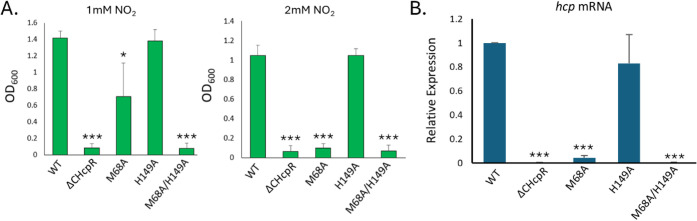
(A) Growth of ΔHcpR*P. gingivalis* complemented with WT and mutant HcpRs (the L156* mutant lacks the DNA-binding domain and serves as a negative control). Complemented strains were grown in 1 mM NO_2_ or 2 mM NO_2_ for 24 h in Tryptic Soy Broth. After incubation, the optical density at 600 nm was used to assess growth. (B) Complemented ΔHcpR*P. gingivalis* strains growing in mid-log phase were exposed to 0.2 mM NO_2_ for 15 min and collected. The level of the *hcp* transcript was assessed via qRT-PCR to assess activity of HcpR mutants. Expression levels of *hcp* mRNA are normalized to the Pg16s rRNA subunit. Asterisks represent *t*-tests with *p* values of * <0.05, **<0.01, and ***<0.001.

To assess the HcpR mutants’ ability to sense reactive nitrogen species, we measured *hcp* transcription *in vivo.* Consistent with the growth studies, we saw a significant decrease of *hcp* transcript in the M68A complemented strain (Figure 4B). However, there was still a modest expression of *hcp*, which explains why the M68A strain exhibited some growth at the lower concentration of nitrite when compared to the L156* negative control. The M68A/H149A double mutant yields *hcp* transcript levels comparable to those of the negative control. The H149A mutant had no significant decrease in *hcp* transcript levels.

To confirm these findings, the M68A, H149A, and M68A/H149A mutant recombinant HcpRs were purified, and the electronic absorption spectra were compared to those of WT HcpR. All proteins were purified under similar conditions and to the same purity as the wild-type protein using cobalt affinity resin. The M68A and H149A single mutants had similar heme-binding capabilities to the WT and retained their coloring with a heme occupancy at 60–70% as measured by the hemochrome assay (Figure 5A). The M68A/H149A double mutant did not bind heme effectively, as evidenced by a loss in color after reconstitution with heme and desalting (the heme reconstitution efficiency was ∼30%) (Figure 5A). The UV–vis spectrum of the M68A/H149A HcpR revealed much less of the heme remained bound, and the heme that was present appeared to be bound nonspecifically, as evidenced by the long, broad Soret band and the lack of α and β bands (Figure 5B).

**Figure 5:**
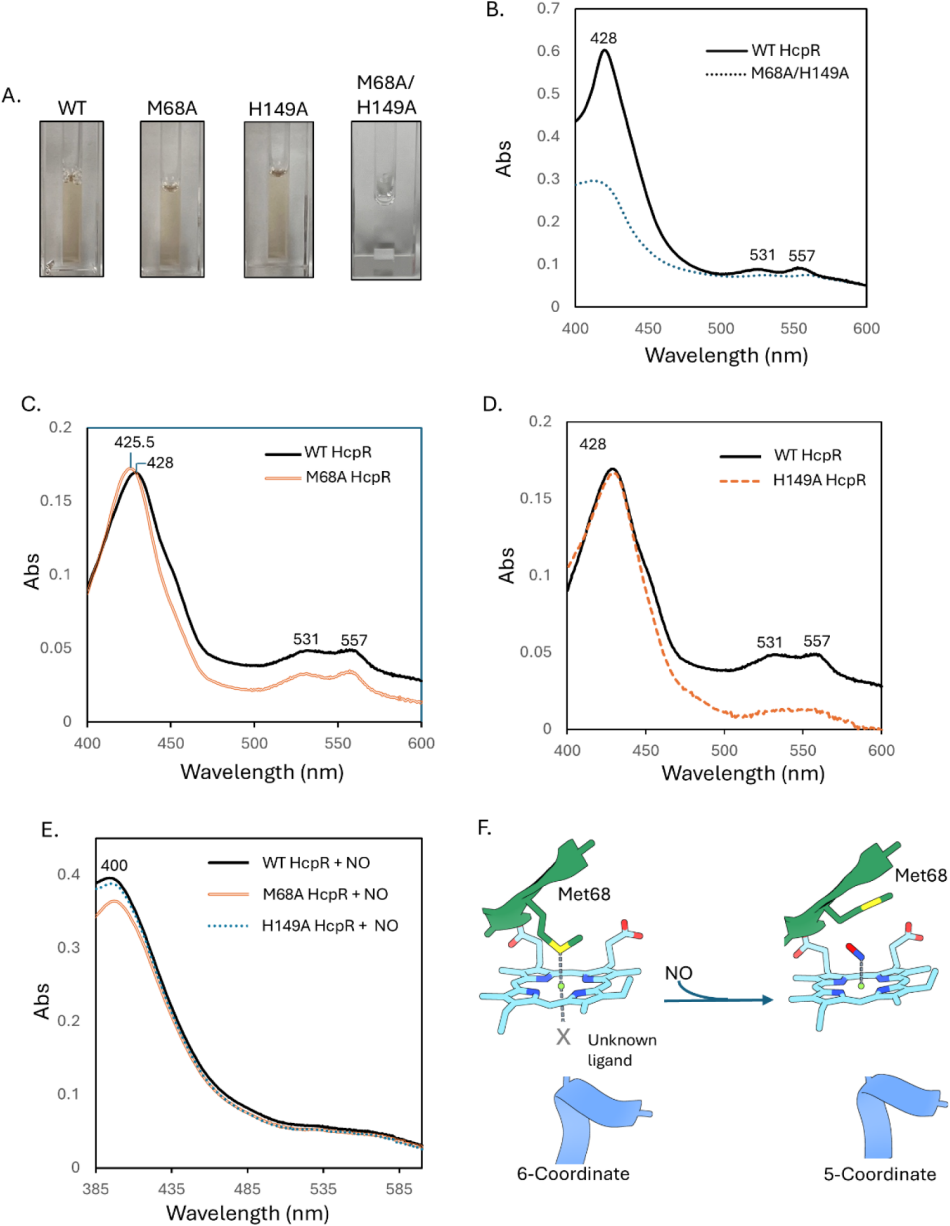
(A) Recombinant tagless and reconstituted WT, M68A, H149A, and M68A/H149A HcpR at 0.25 mg/mL—the amber color of the purified protein indicates the presence of heme in the sample. Amber color in the M68A/H149A mutant is decreased after desalting. The heme occupancy after reconstitution: wildtype-63%; M68A-62%; H149A-60%; M68A/H149A-30% (B) Spectra of the reduced heme-bound WT-HcpR and the M68A/H149A double mutant HcpR at approximately 1 mg/mL. The flattening of the Soret peak in the M68A/H149A HcpR indicates loss of heme binding. (C) Spectra of the reconstituted WT HcpR and M68A HcpR under reduced, anaerobic conditions at 0.2 mg/mL. (D) Spectra of the reconstituted WT HcpR and H149A HcpR under reduced, anaerobic conditions at 0.25 mg/mL. (E) Spectra of the NO-bound WT, M68A, and H149A HcpR under reduced anaerobic conditions. (F) Diagram of the heme coordination before and after the addition of nitric oxide. HcpR is in a 6-coordinate system before the addition of nitric oxide (2-HcpR, 4-N heme). Evidence suggests that Met68 is one of the coordinating ligands. Nitric oxide cleaves both axial bonds from HcpR, and heme exists as a 5-coordinate system (1-NO, 4-N heme). The identity of the 6th axial ligand, possibly His149, is not immediately clear.

The wild-type ferrous HcpR exhibits Soret, α, and β bands at 428, 521, and 557 nm, respectively, under anaerobic conditions, typical of 6-coordinate, low-spin heme proteins (Figure 5C,D). This agrees with previously published resonance Raman spectra of WT HcpR. Ferrous M68A HcpR has Soret, α, and β bands at 425.5, 521, and 557 nm, respectively (Figure 5C). The overall banding pattern is organized like the wild-type HcpR, while the Soret band is slightly blue-shifted by 2.5 nm. Although the α-β bands in the M68A HcpR are located at the same wavelength, there is a flattening of the bands that is indicative of a weaker signal in this region. However, the position of the α-β peaks suggests that HcpR is still in a 6-coordinate system. The blue-shifting of the ferrous Soret band agrees with mutational data observed from ferrous CooA when a coordinating ligand is mutated (His77 in ferrous CooA).^35,36^ Typically, loss of a heme-coordinating residue leads to significant changes in the location of the α-β peaks (i.e., 6-coordinate to a 5-coordinate system). In the other CAP-family heme sensor proteins, mutations in coordinating residues often trigger side chain switching, where the molecular environment around the heme may shift to maintain a 6-coordinate system.^36,37^ The Soret band is sensitive to the overall environment of the heme pocket in heme proteins because it arises from the π electron cloud of the protoporphyrin rings.^38,39^ Thus, the shifted Soret band and weakened α-β bands suggest altered heme orientation in the M68A HcpR. In agreement with both DNR and CooA, the heme environment in HcpR appears to be altered to maintain the 6-coordinate system.^36,37^ Looking into the pocket, a few residues are present that may compensate for Met68 in its absence: Met145 could possibly form an axial bond with an altered heme geometry; Pro100 is in a loop region adjacent to the hydrophobic pocket above Met68 (Figure 3C). However, transcription activation data indicates this alternate coordinating residue is not an adequate replacement for Met68.

H149A HcpR has Soret, α, and β bands located at 428, 521, and 557 nm, respectively (Figure 5D), identical to the WT and indicative of a 6-coordinate, low-spin system, although there is a slight decrease in the intensity of the α-β bands. This suggests that His149 is not a coordinate side chain residue or that the loss of His149 is easily replaced by another side chain in or around the heme pocket. Prior resonance Raman data suggest that one of the coordinating residues in HcpR forms a weak, transient bond that is observable in the polarizing component of the Raman spectrum.^14^ It is possible that His149 is this residue, and its loss is easily compensated by another residue. Still, the expression and heme-binding data of the M68A/H149A double mutant suggest that His149 contributes to heme binding, but it does not play an essential role in the activation mechanism of HcpR, unlike Met68.

NO-bound ferrous HcpR reveals a Soret band at 400 nm and a dimming of the bands in the α-β region. This spectrum was replicated nearly identically by both M68A and H149A mutant HcpRs (Figure 5E). This is consistent with a 5-coordinate gas-bound system whereby both axial ligands of HcpR dissociate and NO is the sole bound axial ligand. This agrees with the resonance Raman spectrum of NO-bound WT HcpR. NO displaces the axial ligands of the heme to form the NO-bound HcpR, creating a 5-coordinate system.^14^ This is likely due to the ability of NO to exert a strong *trans* effect on iron­(II), leading to a long and weak bond to an axial ligand that is easily broken.^40,41^

This data can be summed up in Figure 5F. HcpR adopts a six-coordinate system with Met68 and another axial ligand (possibly His149) acting as axial ligands. The addition of nitric oxide displaces both coordinating ligands, creating a 5-coordinate system. Displacement of these side chains, particularly Met68, may act as the driving switch to activate the HcpR for DNA binding.

### Interdomain Interactions Are Influenced by the Size of the Heme Binding Pocket

In the FL-HcpR, formation of the heme-binding pocket is dependent on the DNA-binding domain of chain A adopting the “ON” conformation, allowing it to form contacts with chain B’s flap region. The effect of this interaction becomes evident when comparing FL-HcpR with ΔCHcpR. The two structures superimpose with an RMSD of 0.56 Å across the full backbone of ΔCHcpR (residues 1–156) (Figure 6A). In ΔCHcpR, the heme-binding location is not obvious, since the typical CRP ligand-binding interface is too small for heme. In FL-HcpR, the flap region of chain B (residues 69–75) differs by ∼13.7 Å (Figure 6B), creating a pocket size difference between the dimer molecules. The pocket of FL-HcpR chain A aligns nearly perfectly with both chains of ΔCHcpR and is significantly smaller (Figure 6A). In both chains of ΔCHcpR and chain A of FL-HcpR, the pocket size is approximately 520 Å^3^. In chain B of FL-HcpR, however, the pocket size increases to approximately 1100 Å^3^ (Figure 6C), better accommodating heme, which has a volume of ∼510 Å^3^.

**Figure 6:**
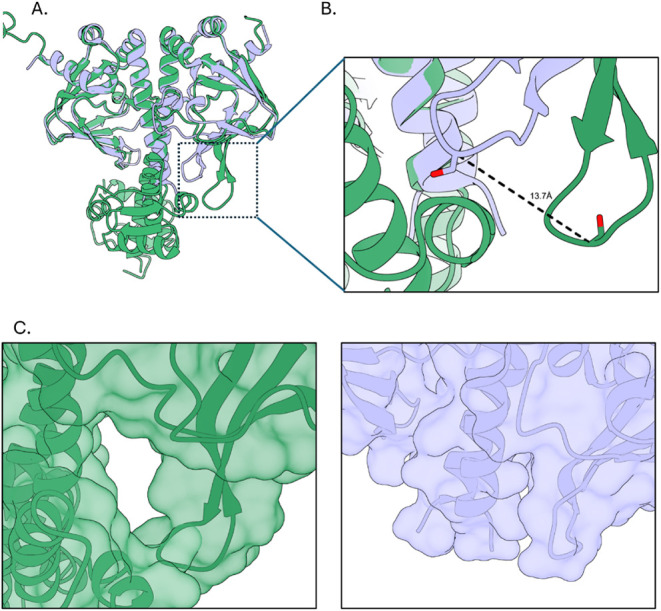
Structural alignment of full-length HcpR and ΔCHcpR highlights the interdomain interactions around the heme pocket. (A) Structural overlay of the full-length HcpR (green) and the C-terminal truncated ΔCHcpR (light purple). The structures overlay with an RMSD of 0.564 Å across the full length of the truncated HcpR (residues 1–156). (B) Zoomed-in view of the boxed region in (A) of the flap region in chain B of HcpR compared to that of chain B in ΔCHcpR. The flap region of HcpR has shifted out by 13.7 Å, enlarging the hydrophobic pocket. (C) Surface view of the boxed region in (A) of the pocket present in the full-length HcpR and the subsequent position and ΔCHcpR.

The DBD conformational differences reflect the hinge flexibility (residues 152–156). In the crystal structure, the DBD orientation is influenced by crystal packing forces (Figure S6), which can stabilize the extended E helix or allow SD-DBD contacts. When the DBD contacts the SD (chain A DBD and chain B SD), this allows a portion of the adjacent homodimer’s N-terminus to occupy the pocket, further stabilizing it (Figure S2A). Notably, the observed SD-DBD interactions are likely facilitated by the increased size of the heme-binding pocket, which allows for interdomain contacts. Previous work established that the ΔCHcpR is capable of binding heme, despite uncertainty regarding the location of the binding site.^14^ This suggests that while the DBD is not required for heme binding, the enlarged pocket in FL-HcpR facilitates SD-DBD interactions. Arg226 of chain A is of particular interest, as it forms polydentate interactions with two backbone residues in the flap loop of chain B and is conserved among the *Porphyromonas* HcpRs. These contacts are reflected in the B-factors of these regions in the two structures: the flap region of chain B in the FL-HcpR is well-ordered with lower B-factors than their corresponding regions in chain A (Figure S7).

## Discussion

In this study, we present the structure of full-length HcpR from *P. gingivalis*. As a phylogenetically distinct sensor-regulator from other heme-based CRP members like DNR and CooA, little is known regarding the nature of heme binding in HcpR. Our full-length structure reveals a series of interdomain interactions that form a hydrophobic pocket in the sensing domain of one of the subunits. Through mutagenesis studies guided by molecular docking and sequence conservation analysis, we propose this site as a putative heme-binding site in HcpR. Notably, it differs from the heme-binding site in CooA, suggesting that HcpR activation may be governed by a distinct mechanism.

The dimer structure shows the C-terminal DNA-binding domains in two different conformations, a property observed in other CRP-family regulators’ X-ray structures (Figure 1).^3^ The “OFF-state”, in accordance with previous studies, was represented by the conformation with an extended E helix in chain B. This conformation positions the DNA-recognition helix in a manner that prevents it from interacting with DNA. Thus, it is likely that in solution, HcpR is dynamic, with the DBDs undergoing open and closed movements. This was demonstrated through SAXS ensemble optimization modeling, which showed that the DNA-binding domain of Apo-HcpR can adopt different conformations in solution.^14^

The two DBD conformations observed in HcpR were also observed in the crystal structures of CooA and CAP. In HcpR, as in CooA, the DNA-binding domain of chain B is in the fully extended conformation with the recognition helix facing in toward the protein in an “off” conformation (Figure 2A).^3^ Chain A’s recognition helix is accessible, though not in the fully outward-facing orientation compared to the structure of CAP bound to DNA.^32^ The dynamic behavior of the two chains reflects the flexibility of the hinge region, around which the DNA-binding domains move, and contributes to the formation of the putative heme-binding pocket (Figure 1C). Upon heme binding, the newly formed interactions between DBD-heme and SD stabilize the “ON” conformation.

Using docking and mutagenesis studies, we identified the putative heme-binding location in a pocket formed by chain A of the DBD and chain B of the SD. Docking implicated Met68 and His149 as heme-coordinating residues (Figure 3B). Both side chains extend into the pocket and are positioned to coordinate the heme. Loss of Met68 severely inhibits HcpR’s ability to activate transcription, decreasing *hcp* expression to <10% (Figure 4B). The minimal remaining hcp expression may explain how the M68A-complemented strain can persist at lower nitrite concentrations. Nitrite is bacteriostatic at these levels, so it is possible that a small amount of HcpR transcriptional activation over time can produce enough *hcp* transcripts to allow for reduced growth. Furthermore, the purified M68A HcpR reconstituted with heme shows differences in its absorption spectrum that are consistent with the altered heme pocket geometry (Figure 5C). Despite maintaining a 6-coordinate system similar to that of the WT HcpR, the M68A spectrum suggests a different orientation of heme in the pocket, possibly allowing another residue to substitute for M68A. Previous studies with CooA and DNR reveal that axial ligand side chain switching can maintain 6-coordinate systems despite deletion of the original residue.^42,43^ However, this substitution cannot replace Met68 in the allosteric network that governs HcpR activation. Based on these data, we have putatively assigned Met68 as one of the heme-coordinating residues in HcpR.

The second coordinating residue is less clear. His149 mutation does not affect HcpR function, and the residue does not appear to be conserved. The H149A HcpR spectrum shows no significant changes in the Soret or α-β bands (Figure 5D). However, the M68A/H149A double mutant HcpR cannot activate transcription like ΔCHcpR, and it has significantly reduced capacity to bind heme, explaining the complete loss of function observed *in vivo* (Figure 5A,B). One possible mechanism involves a water molecule acting as a sixth axial ligand, as observed in heme catalases and peroxidases.^44^ This could benefit a sensor protein such as HcpR, as a water molecule can easily be displaced to allow for substrate binding (NO), and this displacement affects the redox potential of the heme iron. Resonance Raman spectroscopy suggests that one axial bond may be weaker and easily broken, supporting this notion. While His149 itself is not conserved, residues capable of H-bonding are conserved in this position, potentially stabilizing a water molecule.

CRP-family regulators have evolved to sense a wide variety of biologically relevant molecules involved in respiration, stress response, and metabolism. These regulators are based on a common modular design: (1) ligand-binding “jelly roll” motif consisting of β-strands connected to a (2) central α-helix leucine zipper pair linked to a (3) C-terminal HTH DNA-binding domain via a (4) flexible hinge region.^32,45–49^ Ligand binding affects each module, influencing their interactions and stabilizing the DNA-binding domain in an “ON” conformation where recognition helices can access the major groove of target DNA. Despite their similar structural organization, activation depends on specific ligand-induced reorganization of the β-barrel sensing domain and its effect on the allosteric networks between the ligand-binding domain and the DNA-binding domain. Thus, key residues appear to have little conservation among CRP-family members, such as those involved in ligand interaction or cofactor binding.

CRP proteins demonstrate this diversity in their molecular mechanisms. In CAP, cyclic-AMP interacts with residues close to the hinge region, extending the dimerization helix by three turns and creating a coiled-coil motif that stabilizes the positioning of the DNA-binding domain.^50^ In CooA, binding of CO to heme displaces an N-terminal proline residue that frees up the N-terminus to form bridging contacts between the sensing and the DNA-binding domains.^51^ In the halogen sensor CprK, ligand binding reorients the binding pocket, decreasing its volume and affecting the position of key residues in the “flap” region that stabilize the DNA-binding domain through new contacts.^45^

Despite CooA being the best-characterized heme-binding CRP protein, several structural differences distinguish it from HcpR. Although HcpR does have a proline residue near the N-terminus, the two helices adjacent to the N-terminus are well ordered and positioned away from the putative heme-binding site. HcpR’s extended loop region corresponds to CooA’s heme-binding location (Figure 2A). In CooA, this region is shortened to accommodate the heme and is evident in the sequence overlays of HcpR and CooA (Figure S2A). Thus, HcpR’s heme-binding location differs from CooA’s and likely sits “below” it, closer to the C-terminal domain (Figure S5C). CooA’s activation mechanism (the “Velcro” model) involves CO displacement of the N-terminal prolines,^51^ a mechanism that is not compatible with the well-ordered secondary structure at the HcpR N-terminus. Based on the structure and heme-binding studies presented here, it is likely that the HcpR activation mechanism differs from that of CooA.

In the DNR structure, the sensing domain is rotated ∼71° compared to the inactive forms of CooA and HcpR (Figure 2B).^52^ In the DNR’s crystal structure, both monomers are in the “off” conformation, with the extended dimerization helices similar to chain B in FL-HcpR and no contacts between the sensing and DNA-binding domains.^52^ In the absence of SD-DBD interactions and with the angled orientation of the DBD domain, the DNR crystal structure does not appear to have a pocket large enough to accommodate heme. The limited conservation of key heme-binding residues between HcpR and DNR makes mechanistic comparisons difficult.^34,42^

A plausible mechanism for HcpR resembles the pocket rearrangement of CprK upon ligand binding.^45^ NO binding to heme displaces Met68, rearranging the pocket and shifting the flap region along residues 72–75 into a position to make contacts with the DNA-binding domain. These contacts stabilize the DNA-binding domain in a position with the DNA accessible to the recognition helices (Figure S8A). The putative mechanism is supported by several observations: the flap’s proximity to Met68, allowing side chain displacement to influence its position (Figure 1A); the importance of Met68 in HcpR’s heme binding and function (Figures 3, 4, and 5); the conservation of the flap sequence at residues 72–75 (Figure S1A); and the demonstrated flexibility of the flap region when comparing the structures of FL-HcpR and ΔCHcpR (Figure 6B). The solved CRP-family structures in the DNA-bound form all adopt a similar conformation. HcpR adopting a similar conformation would enable Ser72, Lys74, and Gln75 to form hydrogen bonds or salt bridges with the DNA-binding domain.

AlphaFold3 modeling of the DNA-bound HcpR, with a docked heme, further illustrates this mechanism (Figure S8B).^28^ The conserved flap residues form an interface with conserved residues adjacent to the recognition helix (Figure S8C). This includes potential hydrogen bonding between Asn192 and Ser72, as well as the backbone at Gly73, and a potential network of ionic or hydrogen bonding interactions between Lys74/Gln75 and Lys183/Glu184/Asp187. Additionally, Phe189 appears positioned at the entrance to the heme pocket, lending the potential for hydrophobic interactions to occur here. Sequence alignment shows high conservation of both the flap and the “turn” region of the helix-turn-helix motif across *Porphyromonas* and *Prevotella* species (Figure S8C), suggesting their importance in stabilizing the active form.

Further experimental support is needed to confirm this hypothesis. Despite our attempts, we have not yet been able to obtain viable crystals of the heme-protein complex. Additional studies involving the NO binding dynamics and its effects on the heme pocket and DNA binding will help clarify HcpR’s molecular mechanism and the allosteric interactions that govern its function.

## Supplementary Material



## Data Availability

The crystal structure data have been deposited to the Protein Data Bank (PDB) with a title “Crystal Structure of the Transcriptional Regulator HcpR from *Porphyromonas gingivalis*” and assigned the following accession code(s): PDB ID 9DAY.

## Notes

Sequence alignments of HcpR’s found in *Porphyromonas* and *Prevotella* species (Figure S1); additional structural features of full-length HcpR (Figure S2); structural guided sequence alignments of HcpR, CooA, and DNR (Figure S3); comparison of CAP and HcpR structures (Figure S4); extended docking results of heme binding to HcpR (Figure S5); crystal packing in the unit cell of HcpR structure (Figure S6); structural representation of B-factors in HcpR structure (Figure S7); model of full-length HcpR in the active DNA-bound conformation (Figure S8) (PDF)
