# Supplementary material for: Full-Length Structure and Heme Binding in the Transcriptional Regulator HcpR
Source: ACS Omega. 2025 Dec 9;10(50):61179–91. doi: 10.1021/acsomega.5c01735 (PMC12750200; doi:10.1021/acsomega.5c01735)
Supplement: Supplementary file 1 [file ao5c01735_si_001.pdf]

Full-length structure and heme binding in the transcriptional regulator HcpR

Benjamin Ross Belvin[1], Faik N. Musayev[2,3], Carlos R. Escalante[4], Janina P. Lewis[1,4,5 *]

[1]The Philips Institute for Oral Health Research, Virginia Commonwealth University, Richmond, VA 23298

[2]Department of Medicinal Chemistry, Virginia Commonwealth University, Richmond, VA 23298

[3]The Center for Drug Discovery, Virginia Commonwealth University, Richmond, VA 23298

[4]Department of Cellular, Molecular and Genetic Medicine, Virginia Commonwealth University, Richmond, VA 23298

[5]Department of Microbiology and Immunology, Virginia Commonwealth University, Richmond, VA 23298

*Correspondence email: jplewis@vcu.edu

Keywords: Nitrosative Stress, *Porphryomonas gingivalis*, CRP transcriptional regulator, heme binding, HcpR

Figure S1

A.

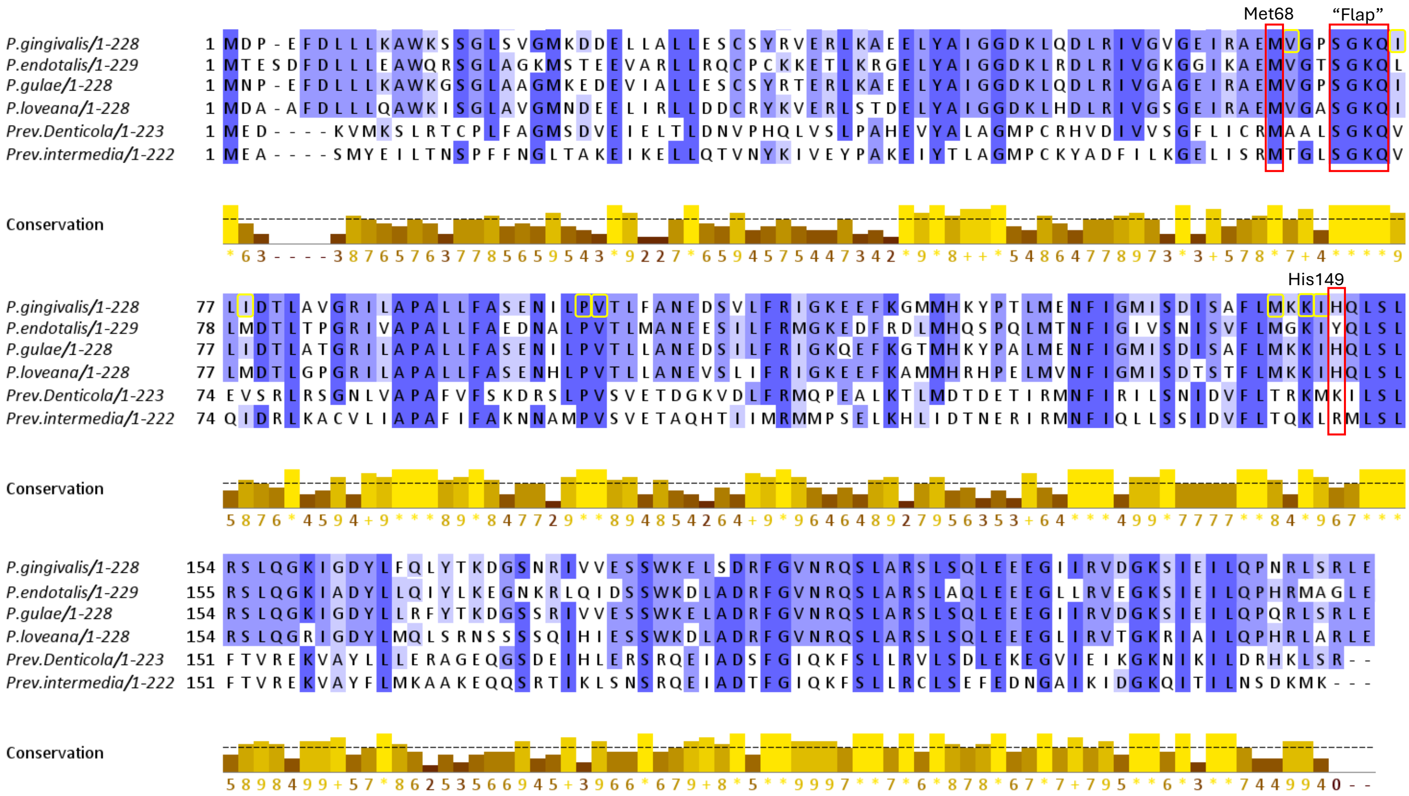

B.

```
>WP_005873453.1 nitrosative stress-sensing transcriptional regulator HcpR
[Porphyromonas gingivalis W83]
MDPEFDLLLKAWKSSGLSVGMKDDELLALLESCSYRVERLKAEELYAIGGDKLQDLRIVGVGEIRAEMVG
PSGKQILIDTLAVGRILAPALLFASENILPVTLFANEDSVLFRIGKEEFKGMMHKYPTLMENFIGMISDI
SAFLMKKIHQLSLRSLQGKIGDYLFQLYTKDGSNRIVVESSWKELSDRFGVNRQSLARSLSQLEEEGIIR
VDGKSIEILQPNRLSRLE
```

Figure S1 - Sequence alinements of HcpR found in *Porphyromonas* and *Prevotella* species. (A) Conservations of residues is graded on a scale of 1 to 11, where 1 indicates no conservation at that residues and 10 (+) near identical conservation and 11 (*) indicates a residue is completely conserved across all sequences. Met68, His149, and the flap region are highlighted in red boxes. Residues that interact with heme in docked structure are in yellow boxes. Sequence accession numbers: *P. endotalis*- WP_314942649 ; *P. gulae*- WP_039417802 ; *P. loveana*- WP_373775352 ; *Prevotella denticola* - WP_029216465; *Prevotella intermedia* - WP_088437813. (B) Sequence of *P. gingivalis* HcpR in FASTA format. Residues mutated in this study, Met68 and His149, are shaded in yellow.

Figure S2

A.

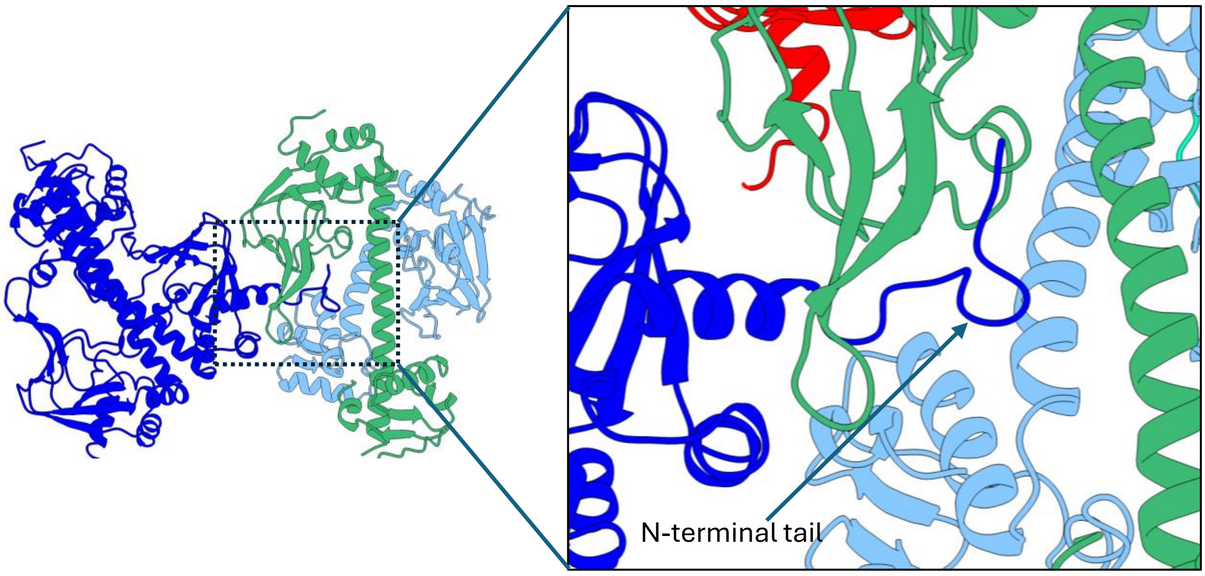

B.

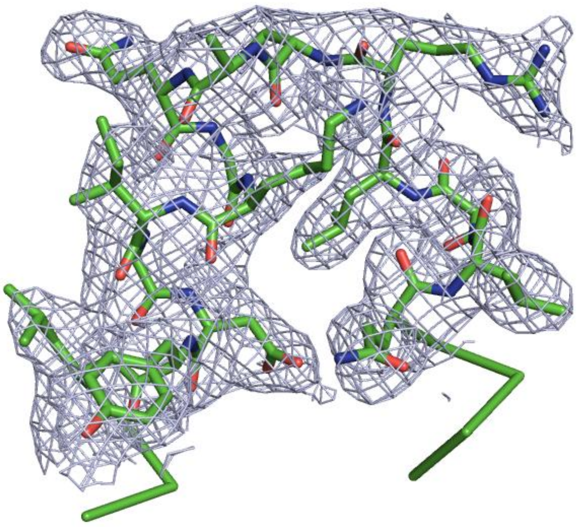

C.

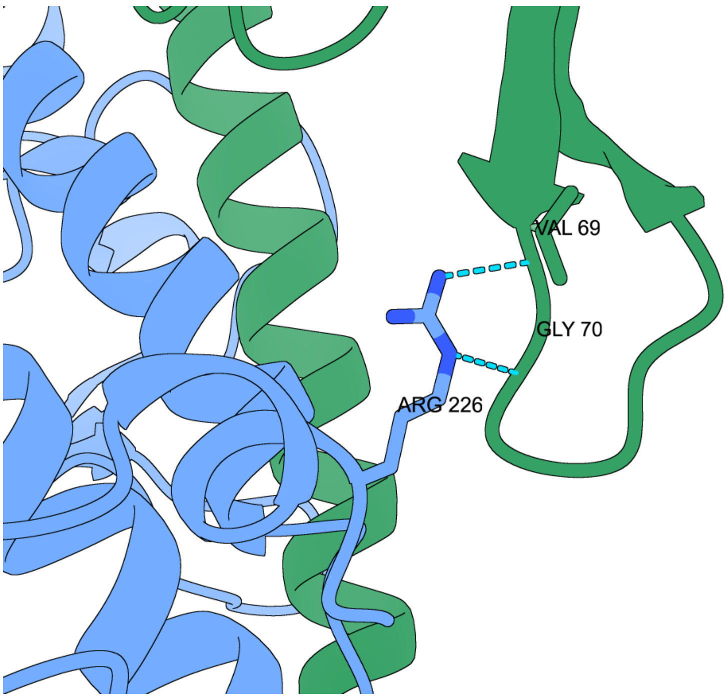

Figure S2 – Structural features of full Length HcpR (A) Presence of the un-cleaved cloning tail from a symmetry related chain (dark blue) can observed in the pocket formed in the homoedimer of chains A (green) and B (light blue). (B) 2fo-fc Electron density map of the hinge region – spanning residues 150-164 (C) Arg226 and the flap region hydrogen bond. Arg226 forms to hydrogen bonds with the flap region along the backbone of Val69 and Gly70.

Figure S3

A.

```
CooA    MPPRFNIANVLLSPDGETFF------------RGFRSKIHAKGSLVCTGE
HcpR    MDPEFDLLLKAWKSSGLSVGMKDDELLALLESCSYRVERLKAEELYAIGG
        * *.*::     ...* :.               .:* :      .*  . *

CooA    GDENGVFVVVDGRLRVYLVGEE-REISLFYLTSGDMFCMHSGCLVEATER
HcpR    DKLQDLRIVGVGEIRAEMVGPSGKQILIDTLAVGRILA---PALLFASEN
        .. :.: :*   *.:*. :** . ::* :   *: * ::.   .*: *:*.

                                               Loss of β6 in HcpR

CooA    -----------TEVRFADIRTFEQKLQTCPSMAWGLIAILGRALTSCMRT
HcpR    ILPVTLFANEDSVLFRIGKEEFKGMMHKYPTLMENFIGMISDISAFLMKK
                   : :   . . *:  ::. *::  .:*.::.   :   *:.

Loop extension in HcpR

CooA    IEDLMFHDIKQRIAGFFIDHANTTGRQTQGGVIVSVDFTVEEIANLIGSS
HcpR    IHQLSLRSLQGKIGDYLFQLYTKDG-----SNRIVVESSWKELSDRFGVN
        *.:* ::.:: :*..:::: .. *      .  : *: : :*::: :* .

CooA    RQTTSTALNSLIKEGYISRQGRGHYT-IPN-LVRLKAAADGDRDDDDD
HcpR    RQSLARSLSQLEEEGIIRVDGKSIEILQPNRLSRLE------------
        **: : :*..* :** *  :*:.     ** * **:
```

B.

```
HcpR   1 MDPEFDLLLKAWKS--SGLSVGMKD-DELLALLESCSYRVERLKAEELYAIGGDKLQDLRIVGVGEIRAEMVG
                        .:.:.::.: ::::::::: : :::::::::::::::::...::::::::::::::::::.
CooA   1 --------------PPRFNIANVLLSPDGETFFR-G-FRSKIHAKGSLVCTGEGDENGVFVVVDGRLRVYLVG

HcpR  71 PSGKQILIDTLAVGRILAPALLFASENILPVTLFANEDSVLFRIGKEEFKGMMHKYPTLMENFIGMISDISAF
           ...:::::::::::: : ..     .:.::: ::::::::::::::::::::::::::::::::::::::
CooA  59 -EEREISLFYLTSGDMF-C-MH-----SGCLVE-ATERTEVRFADIRTFEQKLQTCPSMAWGLIAILGRALTS

HcpR 156 LMKKIHQLSLRSLQGKIGDY
         :::::::::::: ::...
CooA 135 CMRTIEDLMFHD-IKQRI--
```

C.

```
HcpR        ----------------MDPEFDLLLKAWKSSGLSVGMKDDELLALLESCSYRVERLKAEE
DNR         MEFQRVHQQLLQSHHLFEPLSPVQLQELLA--------SSDLVN-LDKGAYVF-------
                            ::*   : *:   :         ..:*:  *:. :* .

HcpR        LYAIGGDKLQDLRIVGVGEIRAEMVGPSGKQILIDTLAVGRILAPALLFASENILPVTLF
DNR         ---RQGEPAHAFYYLISGCVKIYRLTPEGQEKILEVTNERNTFAEAMMFMDTPNYVATAQ
                 *:  : :  :  * ::   : *.*:: :::.    . :* *::* .     .*

HcpR        ANEDSVLFRIGKEEFKGMMHKYPTLMENFIGMISDISAFLMKKIHQLSLRSLQGKIGDYL
DNR         AVVPSQLFRFSNKAYLRQLQDNTPLALALLAKLSTRLHQRIDEIETLSLKNATHRVVRYL
            *   * ***:.:: :   ::.   *   ::. :*       :.:*. ***:.   ::  **

HcpR        FQLYTKDGSN--RIVVESSWKELSDRFGVNRQSLARSLSQLEEEGIIRVDGKSIEILQPN
DNR         LTLAAHAPGENCRVEIPVAKQLVAGHLSIQPETFSRIMHRLGDEGIIHLDGREISILDRE
            : * ::  .:  *: :   : : ::.::.:: ::::* : :* :****::**:.*.**: :

HcpR        RLSRLE
DNR         RLECFE
            **. :*
```

D.

```
HcpR   1 MDPEFDLLLKAWKSSGLSVGMKDDELLALLESC-S-YRVERLKAEELYAIGGDKLQDLRIVGVGEIRAEMVGP
          ..:..::.:::::: :::: :    .::::::::: : ::::::::::::::::::::::::::::::::::::
DNR    1 MEFQRVHQQLLQSH-HLFE-P---LSPVQLQELLASSDLVNLDKGAYVFRQGEPAHAFYYLISGCVKIYRLTP

HcpR  72 SGKQILIDTLAVGRILAPALLFASENILPVTLFANEDSVLFRIGKEEFKGMM-HKYPTLMENFIGMIS--DISA
          .::::::::::::::::::::::::::::::::::::::::::::::::::  ...::..::.....    .
DNR   69 EGQEKILEVTNERNTFAEAMMFMDTPNYVATAQAVVPSQLFRFSNKAYLRQLQDNTPLALALLAKLS-TRL---

HcpR 143 FLMKKIHQLSLRSL-------------------

DNR  139 --------------HQRIDEIETLSLKNATHRV
```

Figure S3 - Sequence alignment of CooA-HcpR and DNR-HcpR. (A & C)The sequence alignment highlights HcpR and CooA (A) and HcpR and DNR (C).In the CooA-HcpR alignment green highlight represents β-sheet structures and teal highlights represents α-helices. (B &D) Structure based alignment using TM-align of the sensing domains of HcpR-CooA (B) and HcpR-DNR (D). Residues acting as axial ligands in CooA (Pro2 and His77) are boxed in red. Met68 in HcpR is highlighted in green. The ":" denotes an aligned residue pairs of d < 5.0 Å, "." denotes other aligned residues

Figure S4

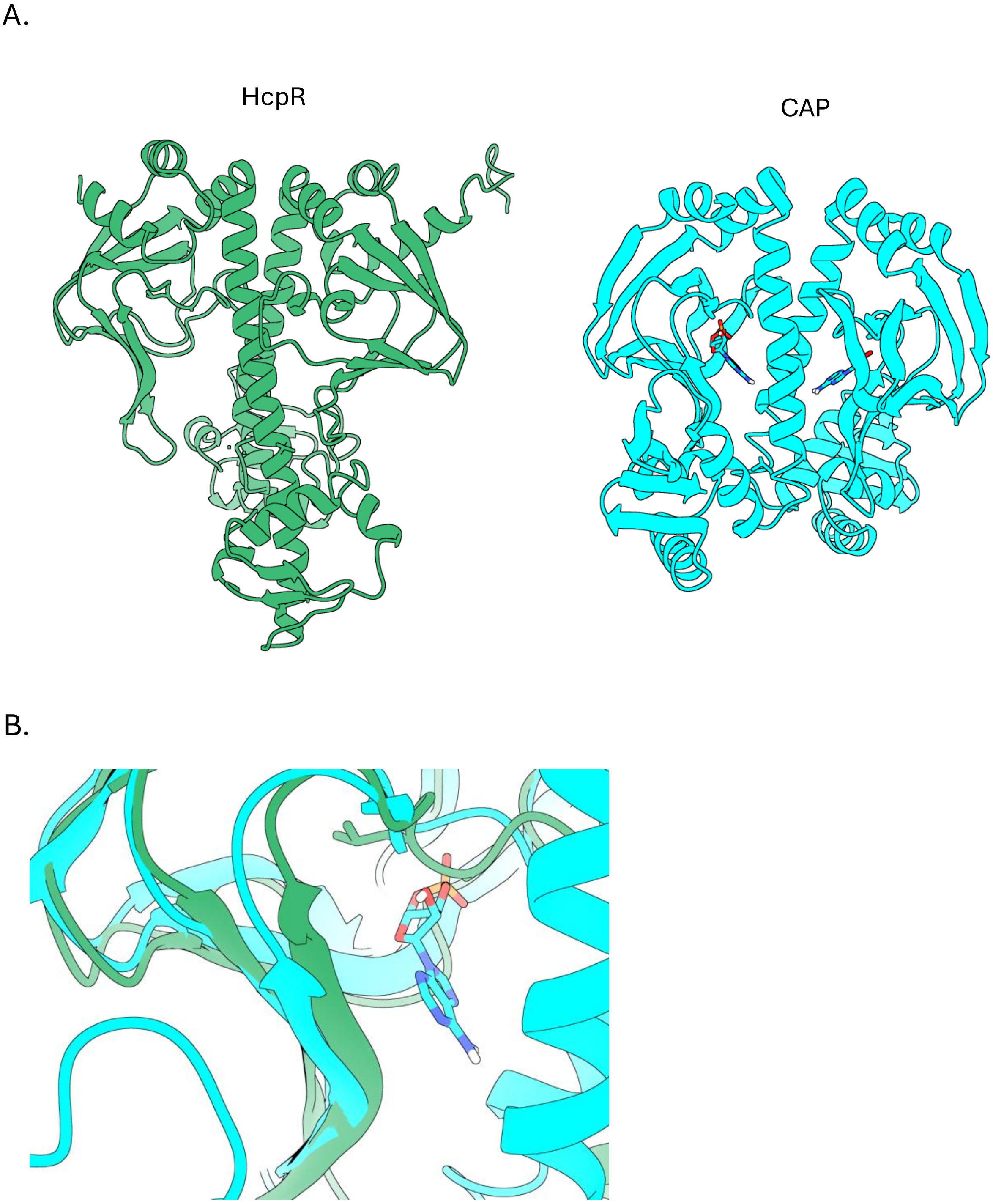

Figure S4 - Comparison of overall fold and conformational differences between HcpR and CAP. (A) View of HcpR (green) and CAP (cyan) dimers depicting the overall organization of each protein. The N-terminal domain of HcpR (extending from residue 1-127) is larger than that of CAP (extending from residues 1-110). (B) Superposition of the HcpR-CAP overlay at the location of cyclic-AMP binding site. The extended loop region of HcpR (89-93) partially occludes this region.

Figure S5

A.

Mode	affinity (kcal/mol)	RMSD l.b. (Å)
1	-8.3	0
2	-8.2	5.01
3	-8.2	6.23
4	-8.1	3.439
5	-8.1	0.905
6	-8.1	2.63
7	-8.0	18.17
8	-7.8	2.97
9	-7.7	2.44

B.

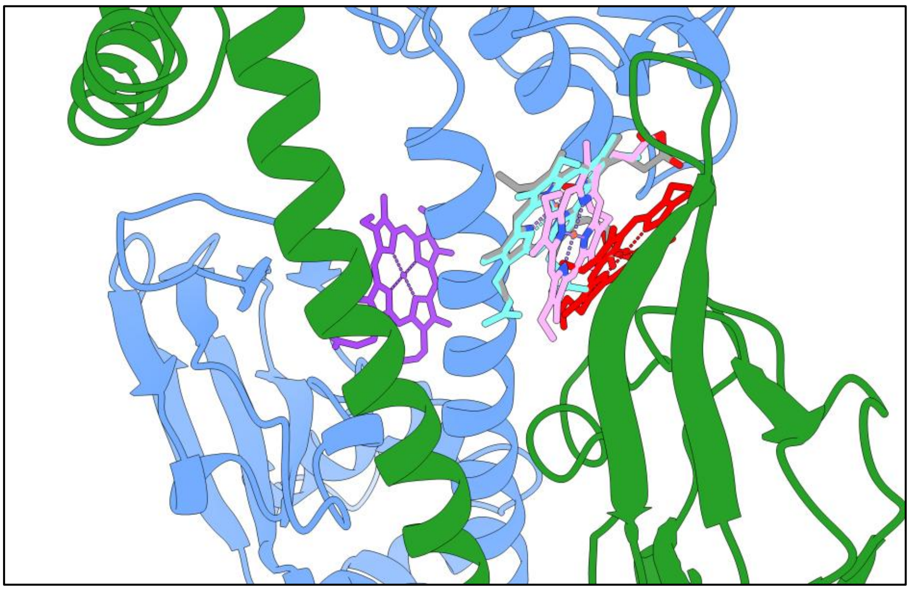

C.

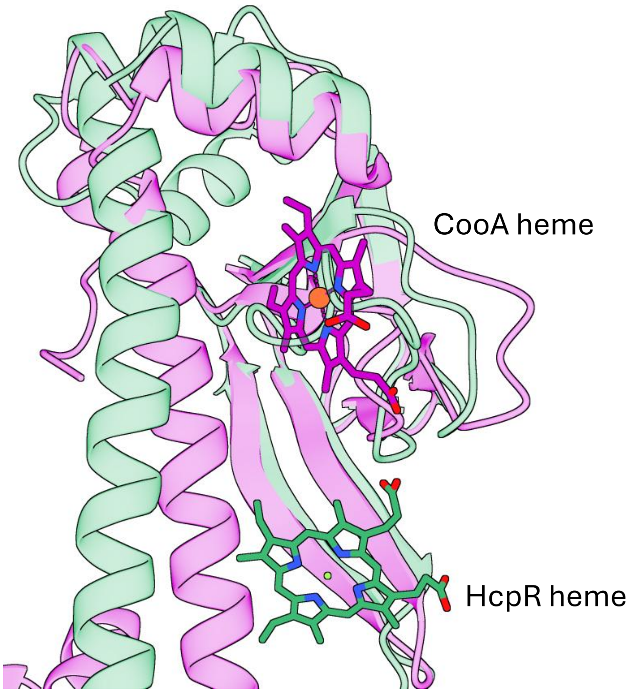

Figure S5. Auto-dock Vina Docking results. (A) Table of results, with the top entry representing the best pose. The RMSD lb reports the shape average fit. The table is color coded to represent the four different poses according to panel B. (B) Ribbon representation of the four different poses heme docking sites. Grey is the best solution, blue are rotations around the plane from the best solution; reds are translation with no iron coordination; purple is a pocket on the BA leucine zipper interface with no heme coordination and pink are positions in the pocket at an angle. (C) Position of heme in CooA (purple) and HcpR (green). The heme binding site identified by docking is “lower” in the sensing domain due to the presence of the extended loop region.

Figure S6

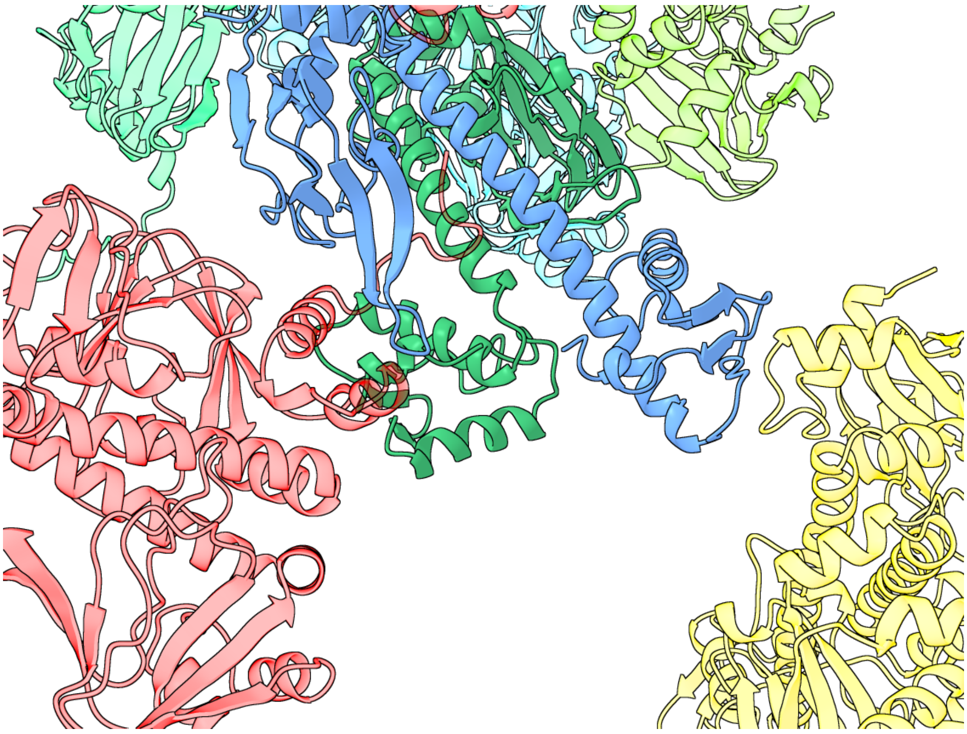

Figure S6 - Crystal packing in the full-length structure of HcpR. The individual homo-dimer represented in blue and green chains with crystal contacts with adjacent homodimers in the crystal lattice. These contacts can either stabilize the dimerization helix (Helix E) in an extended form via contacts with the DNA binding (as in the blue chain A and yellow subunit) or in a form that allows for contacts between the SD and DBD (as in the green chain B and red subunit).

Figure S7

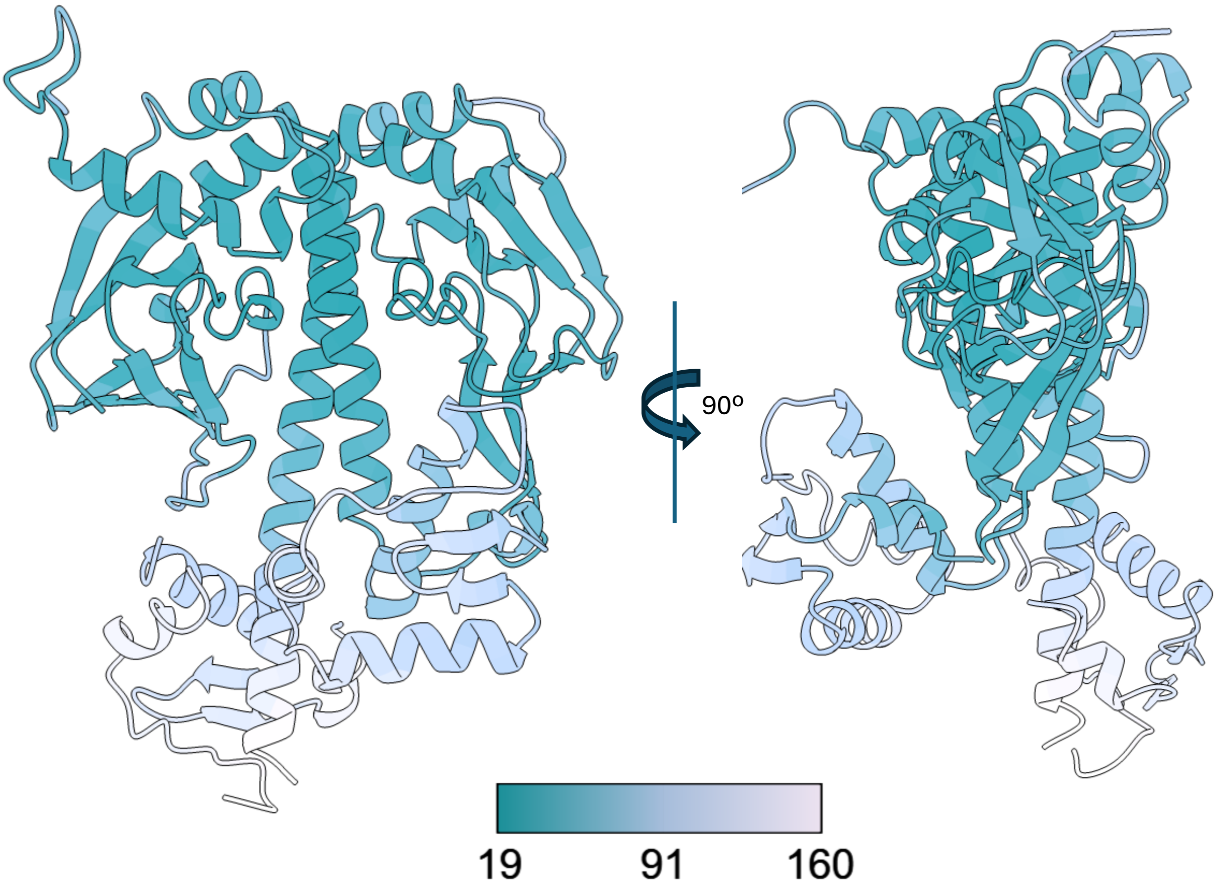

Figure S7 - B-factors in structure of HcpR. Structural representations of B-factors on a scale of 19 (green)-160 (yellow).

Figure S8

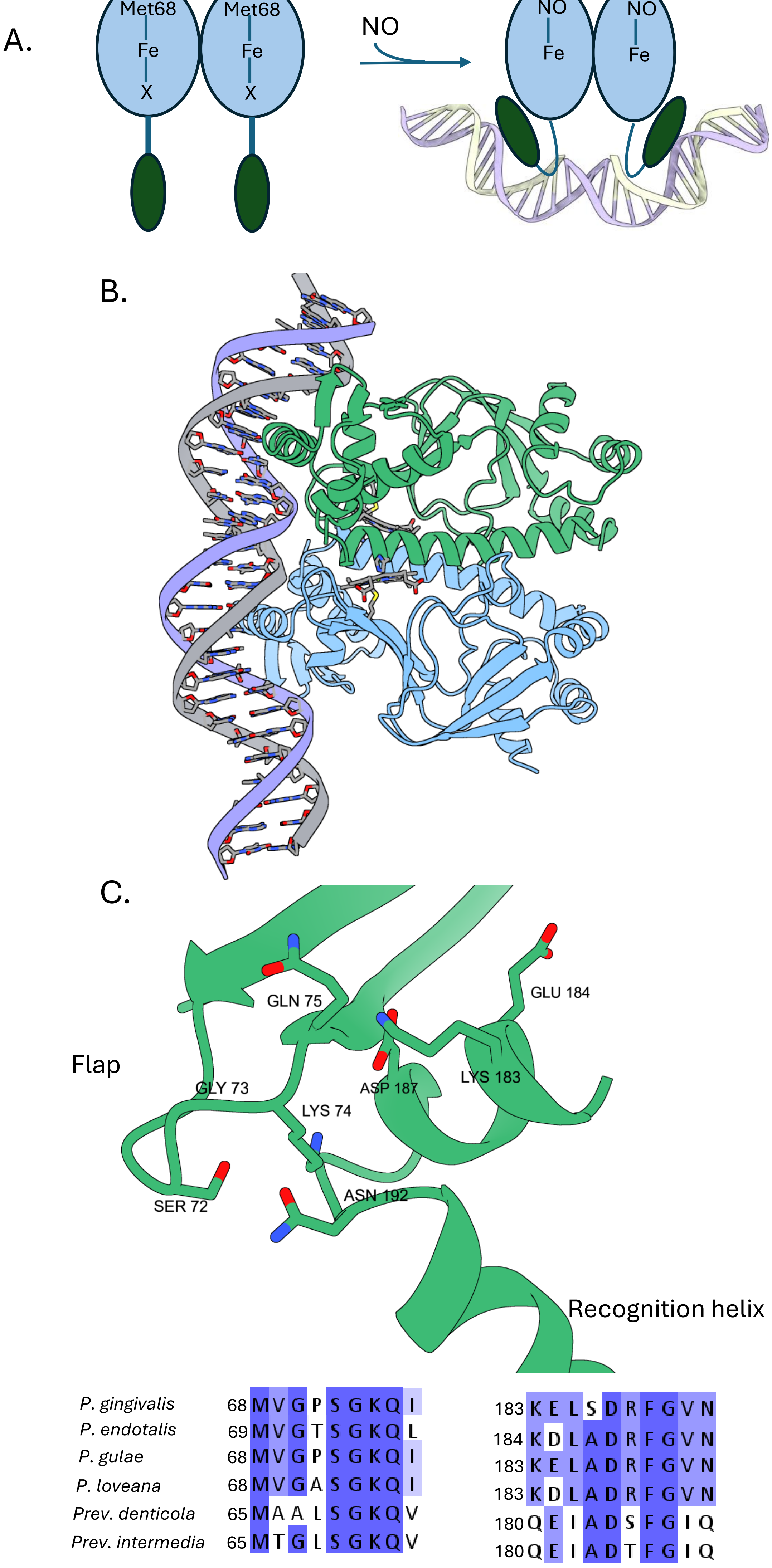

Figure S8 - Model of the full length HcpR structure in the active conformation. (A) Overview of the putative HcpR mechanism of activation. In the off state, Met68 remains bound along with a yet undescribed secondary axial ligand. The DNA binding domains are in an “off” state where the dimerization helices are not accessible to DNA. NO binding displaces both axial ligands from the heme. This causes an allosteric network to cascade, leading to the stabilization of the DNA binding domains in an “on” confirmation with their recognition helices accessible to the major groove of DNA. (B) Model of the homo-dimer in the active conformation bound to DNA. (C) Conserved residues that form the interface between the flap along residues 72-75 and the DBD along residues 183-192 with the sequence alignments of each region. Sequence accession numbers: *P. endotalis*- WP_314942649 ; *P. gulae*- WP_039417802 ; *P. loveana*- WP_373775352 ; *Prevotella denticola* - WP_029216465; *Prevotella intermedia* - WP_088437813.
